# Repeated naloxone-induced morphine withdrawal alters blood brain barrier and blood spinal cord barrier integrity in mice

**DOI:** 10.1186/s13041-025-01231-9

**Published:** 2025-07-07

**Authors:** Yuta Kohro, Craig E. Brown, Tuan Trang

**Affiliations:** 1 Faculty of Veterinary Medicine, University of Calgary, Calgary, AB Canada; 2 Department of Physiology and Pharmacology, Hotchkiss Brain Institute, University of Calgary, Calgary, AB Canada; 3 Department of Molecular and System Pharmacology, Graduate School of Pharmaceutical Sciences, Kyushu University, Fukuoka, Japan; 4 Division of Medical Sciences, University of Victoria, Victoria, BC Canada; 5 Department of Psychiatry, University of British Columbia, Vancouver, BC Canada

**Keywords:** Opioid, Blood brain barrier, Blood spinal cord barrier, Brainstem, Opioid withdrawal

## Abstract

Passage of molecules across the central nervous system is tightly regulated by the blood-brain barrier (BBB) and blood-spinal cord barrier (BSCB), which restrict entry of many substances, including opioid medications. Here, we examined the effects of opioid withdrawal on BBB and BSCB integrity by measuring extravascular levels of peripherally injected dyes – Evans Blue (high molecular weight) and sodium fluorescein (NaFl, low molecular weight) – in the brain and spinal cord. In morphine-dependent male and female mice, repeated naloxone challenge induced robust withdrawal behaviors concomitant with region specific dye extravasation. In a fixed dose morphine paradigm, Evans Blue extravasation was highest within the cortex, hippocampus, cerebellum, and brainstem (pons and medulla) in male mice, and in the hypothalamus in female mice. By contrast, NaFl extravasation remained unchanged in both sexes. In an escalating dose morphine paradigm, Evans Blue extravasation was most prominent in the brainstem (pons and medulla) of both sexes, as well as in the lumbar of male mice and cervical spinal cord of female mice. NaFl extravasation in these regions was unchanged in male but reduced in female mice. These findings suggest that repeated opioid withdrawal alters permeability of the BBB and BSCB in discrete regions of the brain and spinal cord.

## Introduction

The blood-brain barrier (BBB) and blood-spinal cord barrier (BSCB) are critical in maintaining structural and functional homeostasis of the central nervous system (CNS). Comprised of an endothelial cell monolayer, the BBB and BSCB interact with perivascular pericytes, microglia, astrocytes, and neurons which together form the neurovascular unit [1–3]. This vascular interface restricts passage of cells and molecules across the CNS, protecting the health and activity of neural networks within the brain and spinal cord. However, barrier integrity can be compromised in diseases such as epilepsy, stroke, neurodegeneration, and following injury [1–3]. Opioid medications and a variety of drugs with addiction liability also disrupt barrier permeability [4, 5]. Indeed, polydrug use in people with substance use disorder is associated with metabolic and vascular changes that compromise BBB integrity [6, 7].

Opioid entry into the CNS is modulated by influx and efflux transporters, such as P-glycoprotein [8–10]. This regulated access influences the rate and extent of opioid distribution; it is also a determinant of opioid analgesia affecting centrally mediated adverse opioid effects, including respiratory depression, reward, and dependence [11, 12]. Opioid dependence can develop with chronic use and presents as withdrawal symptoms on cessation or decreasing opioid use [13, 14]. To avoid or alleviate these symptoms, people often continue to use opioids, experiencing cycles of withdrawal which make it difficult for people to stop opioid use [15, 16]. The cycle of opioid use and withdrawal has adverse physiological, cognitive, and mental outcomes associated with opioid dependence and addiction [17].

In morphine-dependent rodents, abrupt opioid cessation elicits withdrawal behaviors associated with an increase in brain and spinal dye extravasation [18]. This increased central permeability suggests that the BBB and BSCB are compromised during opioid withdrawal. In mice, we asked whether integrity of the BBB and BSCB is affected by repeated opioid withdrawal, akin to the cycles of withdrawal followed by continued opioid use experienced by people. Here, we test the hypothesis that repeated naloxone-induced withdrawal differentially affects permeability in discrete brain and spinal cord regions.

## Results

### Repeated exposure to naloxone with a fixed dose of morphine induces morphine withdrawal

**Fig. 1:**
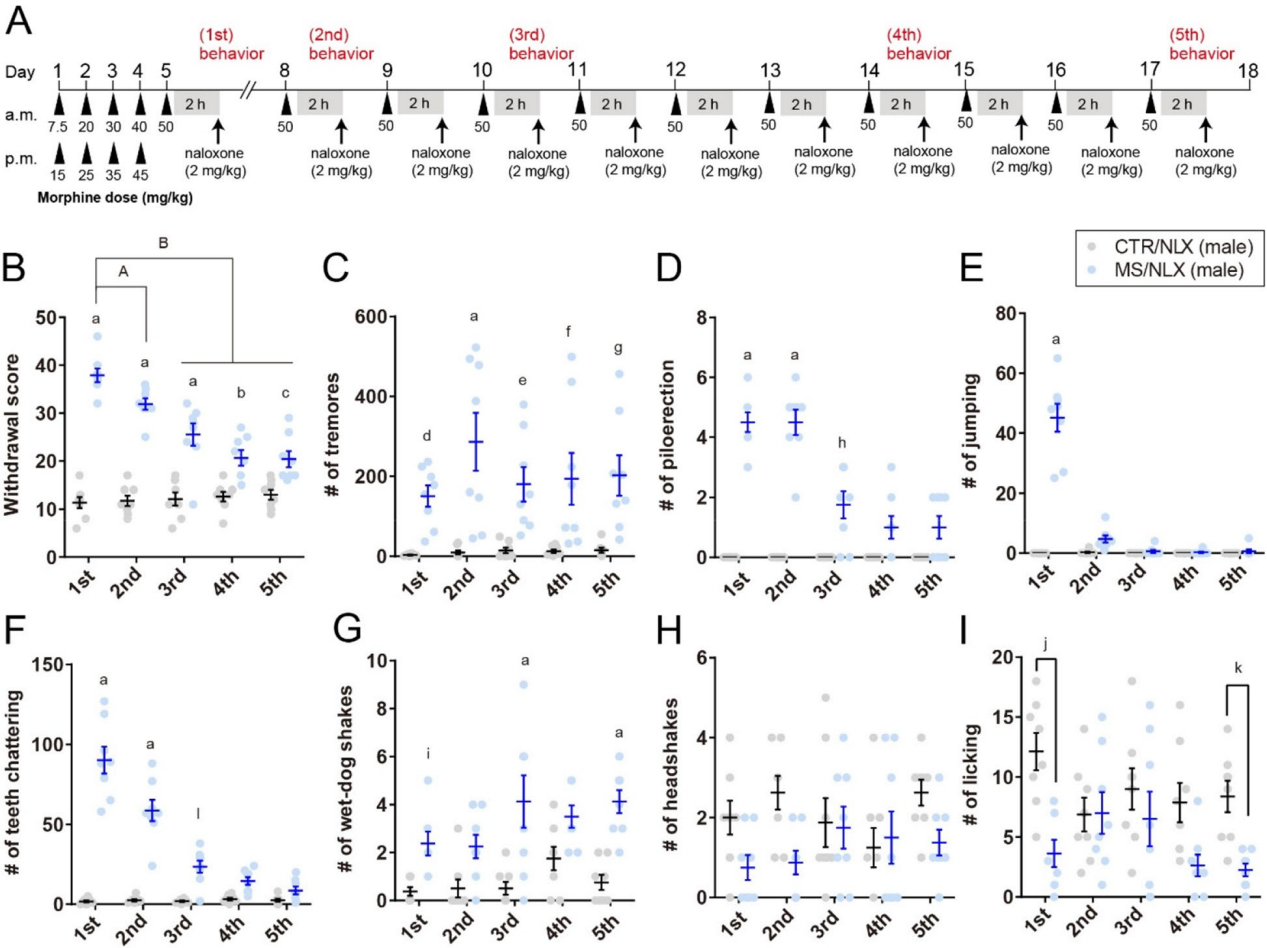
Naloxone-precipitated withdrawal in morphine-dependent male mice. **A**, Schematic depicting morphine sulfate (MS) dosing. Mice were rendered morphine dependent over 5 days and then treated with a fixed dose of morphine (50 mg/kg) with subsequent naloxone (NLX) precipitated morphine withdrawal. Control group (CTR) were treated with saline and NLX. **B**, Cumulative withdrawal scores in male mice after single morphine withdrawal or repeated once, three-, seven- and ten-times withdrawal from a fixed dose of morphine (1st, 2nd, 3rd, 4th or 5th, respectively) (*n* = 8 mice).** C-I**, The number of tremors (**C**), piloerection (**D**), jumping (**E**), teeth chattering (**F**), wet-dog shakes (**G**), headshakes (**H**) and licking (**I**) in male mice after single- or repeated fixed dose of morphine withdrawal (*n* = 8 mice). a, *P* < 0.0001; b, *P* = 0.001; c, *P* = 0.0028; d, *P* = 0.0412; e, *P* = 0.00159; f, *P* = 0.0066; g, *P* = 0.0047; h, *P* = 0.0002; i, *P* = 0.0418; j, *P* = 0.0007; k, *P* = 0.025; l, *P* = 0.0008 versus CTR/NLX, A, *P* = 0.0009; B, *P* < 0.0001 versus MS/NLX single morphine withdrawal. Data show the mean ± SEM

To establish physical dependence, adult male and female C57BL/6 mice received escalating doses of morphine (7.5–50 mg/kg) over five days [13, 19]. Beginning on day 8, mice were maintained on a fixed morphine dose (50 mg/kg) through day 18 to model repeated opioid use without further dose escalation (Fig. 1A). In male mice, administration of the opioid receptor antagonist naloxone elicited robust withdrawal behaviors in morphine-treated compared to saline-treated controls (Fig. 1B). Observed withdrawal signs included tremors, piloerection, jumping, teeth chattering, and wet-dog shakes (Fig. 1C-G). A composite withdrawal score, representing the constellation of these behaviors, was significantly higher in morphine-treated mice. However, headshakes did not reach statistical significance, and bouts of licking were reduced (Fig. 1H-I). Following the first naloxone-induced withdrawal episode, a fixed dose of morphine (50 mg/kg) administration continued daily from days 8-17. Naloxone challenges were conducted 2 h after morphine treatment on each of these days [20]. Repeated naloxone challenges elicited higher composite withdrawal scores in morphine-treated than in saline-treated control mice (Fig. 1A and B).

**Fig. 2:**
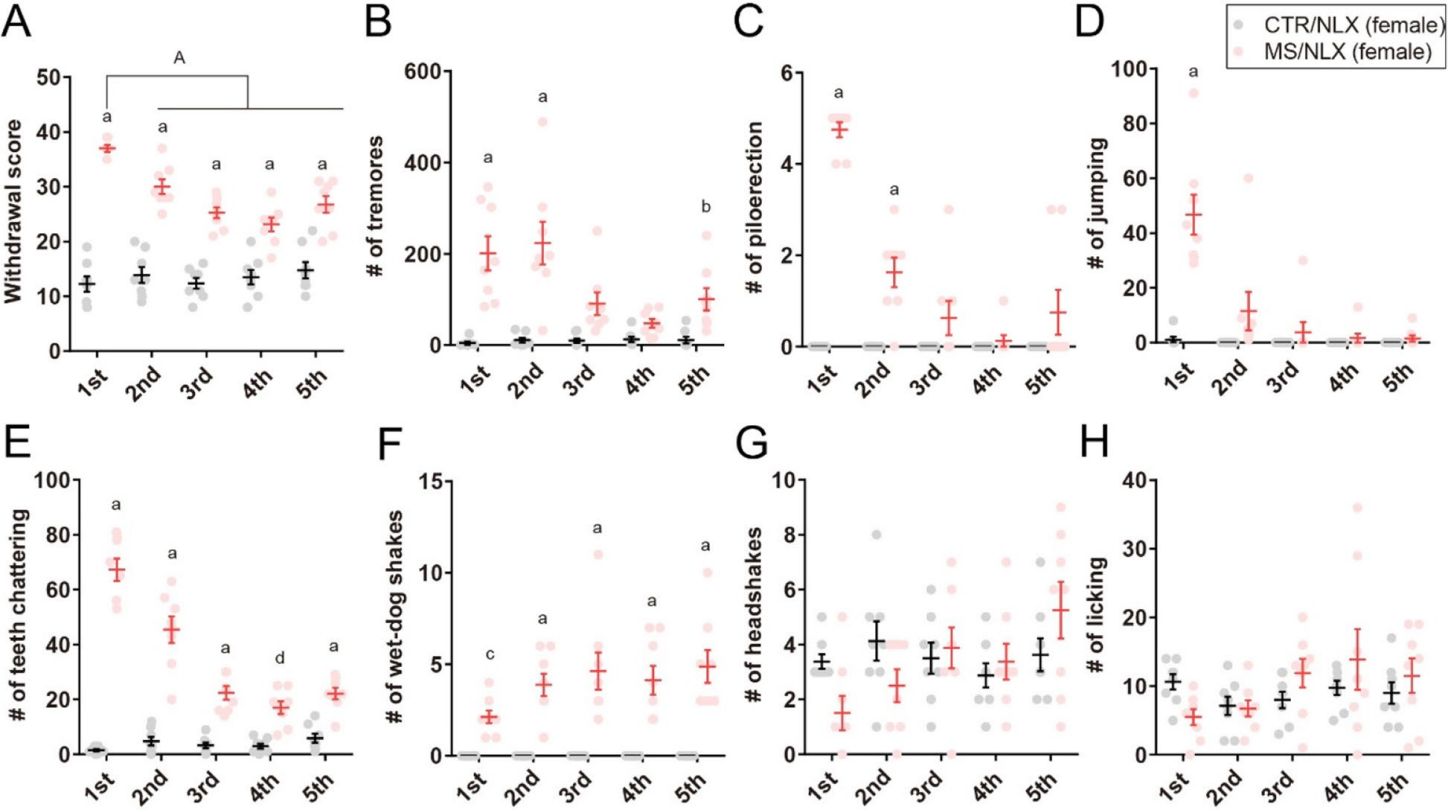
Naloxone-precipitated withdrawal in morphine-dependent female mice. **A**, Cumulative withdrawal scores in female mice after single- or repeated withdrawal from a fixed dose of morphine (*n* = 8 mice). **B–H**, The number of tremors (**B**), piloerection (**C**), jumping (**D**), teeth chattering (**E**), wet-dog shakes (**F**), headshakes (**G**) and licking (**H**) in female mice after single- or repeated withdrawal from a fixed dose of morphine (*n* = 8 mice). a, *P* < 0.0001; b, *P* = 0.0308; c, *P* = 0.0364; d, *P* = 0.0009 versus CTR/NLX, A, *P* < 0.0001 versus MS/NLX single morphine withdrawal. Data show the mean ± SEM

In female mice, naloxone similarly precipitated robust withdrawal behaviors across repeated challenges (Fig. 2A-H). Interestingly, in both sexes, the cumulative withdrawal scores declined following the second naloxone challenges compared to the first (Figs. 1B and 2A). Notably, the frequency specific signs, such as piloerections, jumping and teeth chattering, gradually decreased with successive withdrawal episodes (Figs. 1D-F and 2C-E). These results indicate that repeated naloxone challenges reliably induce withdrawal behaviors in morphine-dependent male and female mice, although the severity of these behaviors diminishes with repeated exposure.

### BBB and BSCB are disrupted by repeated withdrawal from a fixed dose of morphine

**Fig. 3:**
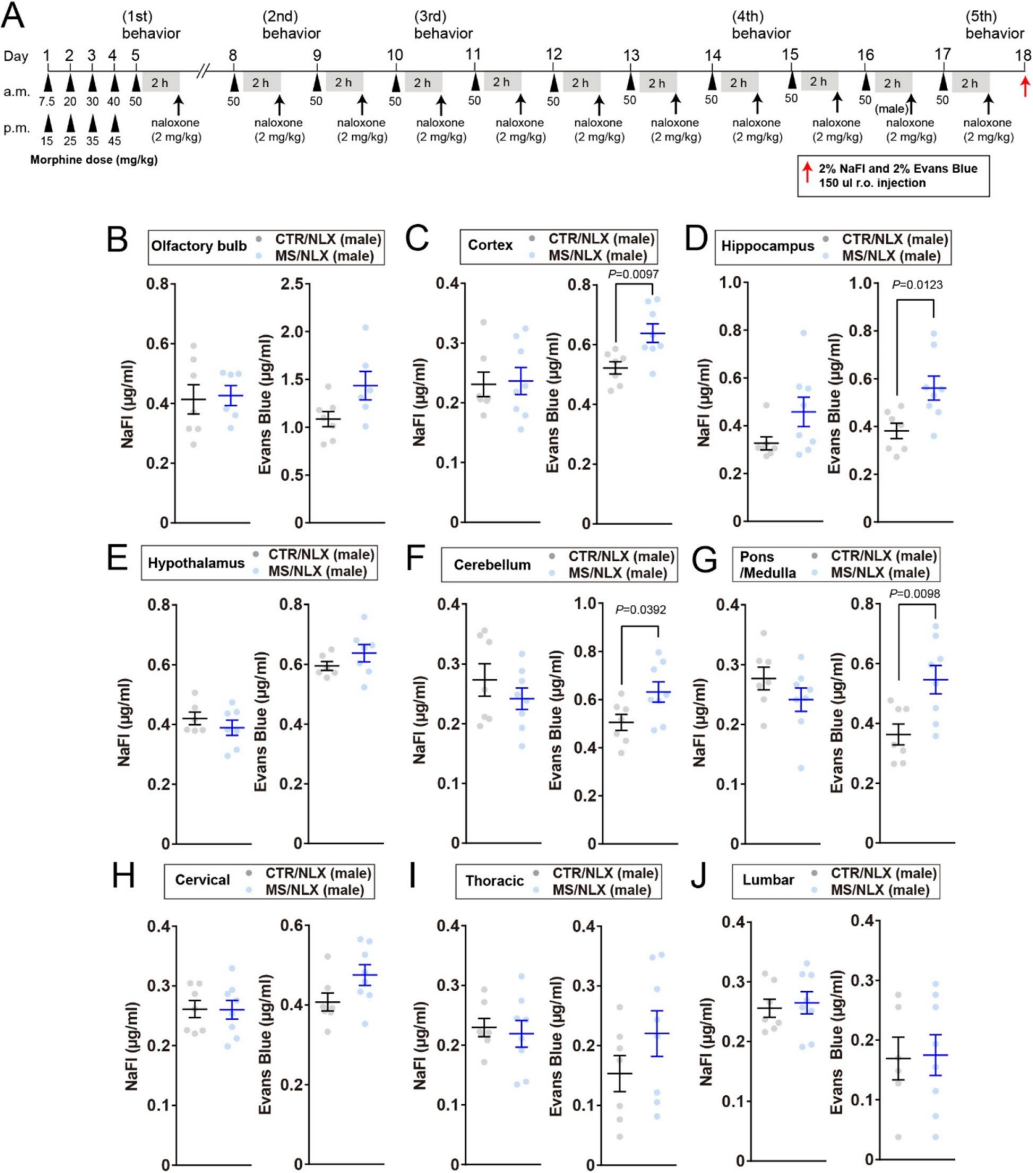
Increased Evans Blue extravasation in the cortex, hippocampus, cerebellum and pons/medulla of morphine-dependent male mice. **A**, Schematic timeline for morphine dosing, naloxone precipitated morphine withdrawal and injection of dye tracers. Dye tracers are retro-orbitally injected 24 h after the last morphine withdrawal. **B–J**, NaFl and Evans Blue permeabilities into the olfactory bulb (**B**), cortex (**C**), hippocampus (**D**), hypothalamus (**E**), cerebellum (**F**), pons/medulla (**G**), cervical (**H**), thoracic (**I**) and lumbar (**J**) of male mice after repeated withdrawal from a fixed dose of morphine (*n* = 6–8 mice). Data show the mean ± SEM

**Fig. 4:**
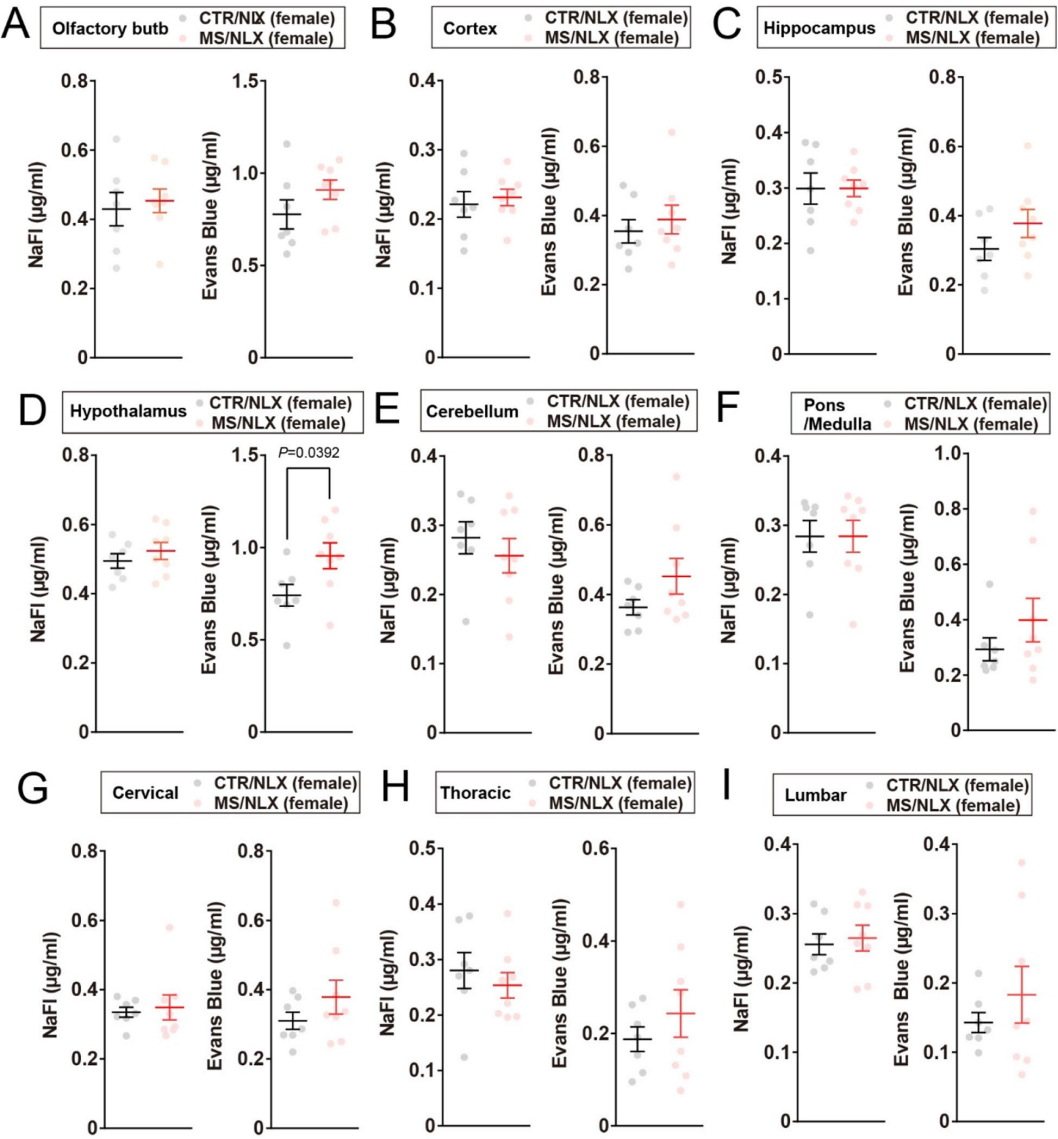
Increased Evans Blue extravasation in the hypothalamus of morphine-dependent female mice. **A-I**, NaFl and Evans Blue permeabilities into the olfactory bulb (**A**), cortex (**B**), hippocampus (**C**), hypothalamus (**D**), cerebellum (**E**), cervical (**G**), thoracic (**H**) and lumbar (**I**) of female mice after repeated withdrawal from a fixed dose of morphine (*n* = 7–8 mice). Data show the mean ± SEM

To examine whether morphine withdrawal affects BBB and BSCB permeability, mice were peripherally injected with either a high molecular weight dye (Evans blue, ~ 68 kDa when bound with albumin) or a low one (sodium fluorescein (NaFl); 376 Da) 24 h after morphine withdrawal on day17 (Fig. 3A). Increased dye penetration into CNS tissues serves as a surrogate measure of a compromised BBB and BSCB integrity [3]. In male mice, repeated morphine withdrawal increased Evans Blue extravasation in the cortex, hippocampus, cerebellum and pons and medulla region of the brainstem (Fig. 3C, D, F and G**)**. With regard to other regions such as the olfactory bulb, hypothalamus, cervical, thoracic and lumbar spinal cord of male mice, Evans Blue extravasation was similar between morphine withdrawn and control mice (Fig. 3B, E, H, I and J). By contrast, Evans Blue extravasation was increased only in the hypothalamus but not other regions of female mice (Fig. 4A-I). NaFl permeability was unchanged in all regions of both sexes after repeated morphine withdrawal (Figs. 3B-J and 4A-I). These results indicate that repeated morphine withdrawal disrupts the discrete regions of BBB and BSCB in a sex-dependent manner.

### Repeated exposure to naloxone with escalating doses of morphine induces morphine withdrawal

**Fig. 5:**
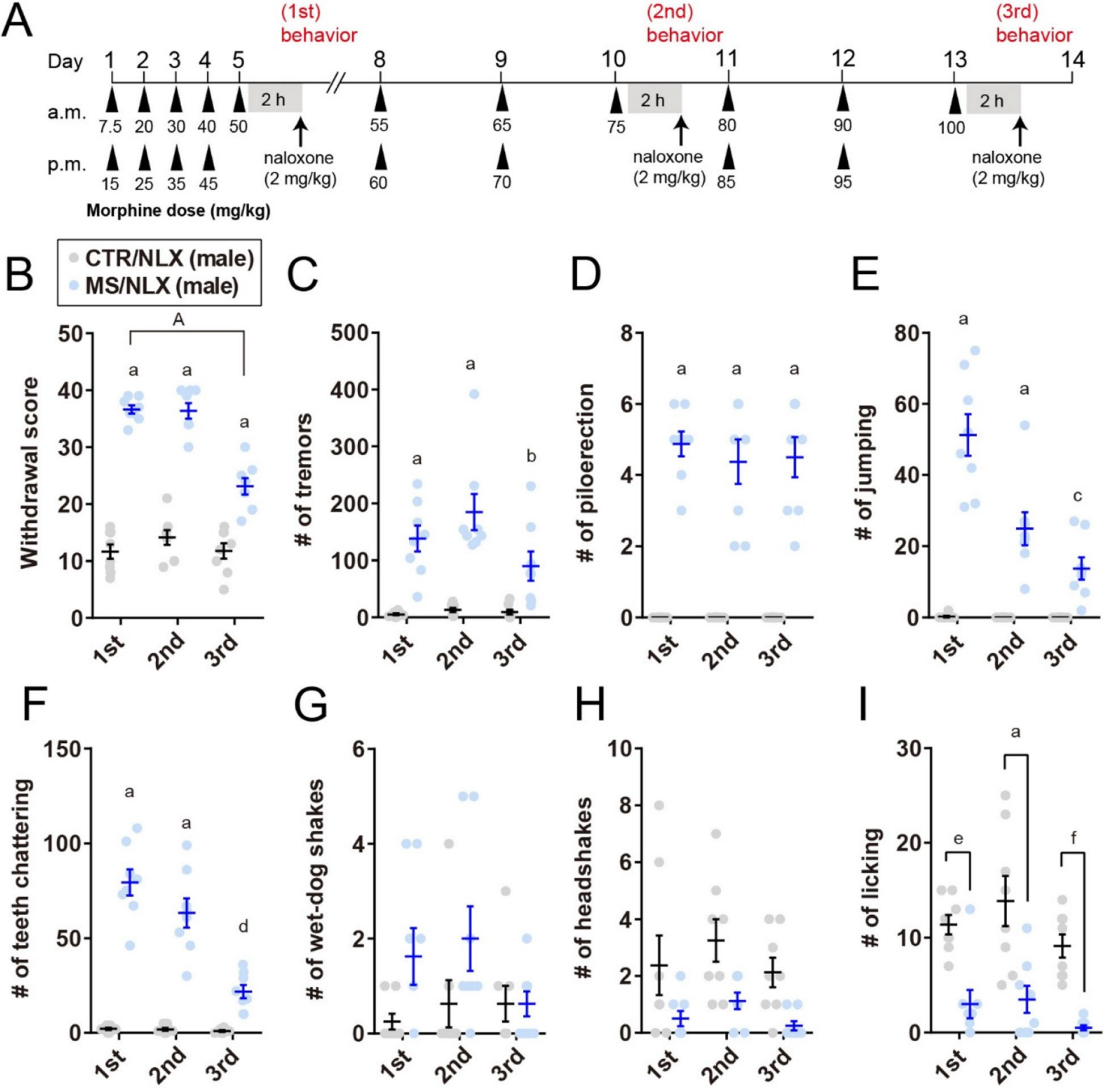
Repeated naloxone challenge in male mice with escalating doses of morphine induces withdrawal behaviors. **A**, Schematic depicting morphine dosing, naloxone precipitated morphine withdrawal, and behavioral test for escalating doses of morphine repeated withdrawal. Control group were treated with saline and naloxone. **B**, Cumulative withdrawal scores in male mice after first, second and third morphine withdrawal (*n* = 8 mice). **C-I**, The number of tremors (**C**), piloerection (**D**), jumping (**E**), teeth chattering (**F**), wet-dog shakes (**G**), headshakes (**H**) and licking (**I**) in male mice after first, second and third morphine withdrawal (*n* = 8 mice). a, *P* < 0.0001; b, *P* = 0.0146; c, *P* = 0.0163; d, *P* = 0.0061; e, *P* = 0.001; f, *P* = 0.0007 versus CTR/NLX, A, *P* < 0.0001 versus MS/NLX single morphine withdrawal. Data show the mean ± SEM

**Fig. 6:**
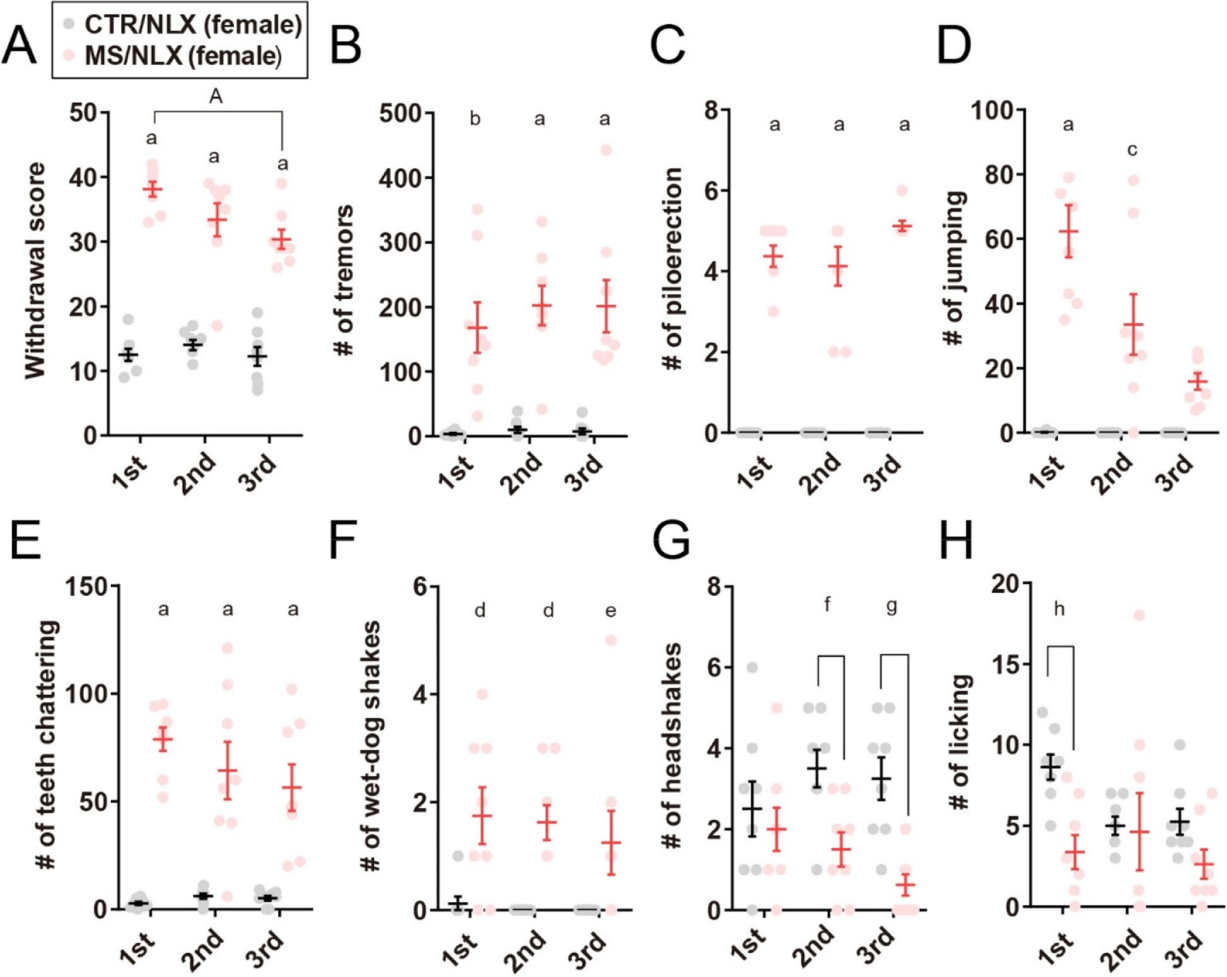
Repeated naloxone challenge in female mice with escalating doses of morphine induces withdrawal behaviors. **A**, Cumulative withdrawal scores in female mice after first, second and third naloxone challenge (*n* = 8 mice). **B-H**, The number of tremors (**B**), piloerection (**C**), jumping (**D**), teeth chattering (**E**), wet-dog shakes (**F**), headshakes (**G**) and licking (**H**) in female mice after first, second and third naloxone challenge (*n* = 8 mice). a, *P* < 0.0001; b, *P* = 0.0002; c, *P* = 0.0001; d, *P* = 0.0066; e, *P* = 0.0483; f, *P* = 0.0208; g, *P* = 0.0017; h, *P* = 0.0134 versus CTR/NLX, A, *P* = 0.0044 versus MS/NLX single morphine withdrawal. Data show the mean ± SEM

Dose escalation is a major concern in individuals struggling with the cycle of opioid use and withdrawal [17]. To model this, mice were initially treated with escalating doses of morphine (7.5–50 mg/kg) over five days (as described above). After a naloxone challenge, morphine dosing resumed at 55 mg/kg on day 8 and was progressively increased, reaching a final dose of 100 mg/kg by day 13 followed by naloxone precipitated withdrawal (Fig. 5A**)**. Both the second and third challenges in male and female mice resulted in composite withdrawal scores that were higher in morphine-treated as compared to saline-treated control mice (Figs. 5B and 6A). In both sexes, the cumulative withdrawal score was significantly lower during the third naloxone challenge compared to earlier challenges (Figs. 5B and 6A**)**. The observed withdrawal behaviors included tremors, piloerection, jumping, and teeth chattering in both sexes (Figs. 5C-F and 6B-E). Furthermore, wet-dog shakes increased in female mice but did not reach statistical significance in male mice (Figs. 5G and 6F). In contrast, licking and headshakes significantly decreased or tended to decrease in both sexes (Figs. 5H, I and 6G, H). Thus, similar with repeated withdrawal from a fixed dose of morphine, these results indicate that repeated naloxone challenges with escalating doses of morphine produce robust morphine withdrawal behaviors in both male and female mice.

### BBB and BSCB permeability are altered in a size-selective manner by repeated withdrawal from escalating doses of morphine

**Fig. 7:**
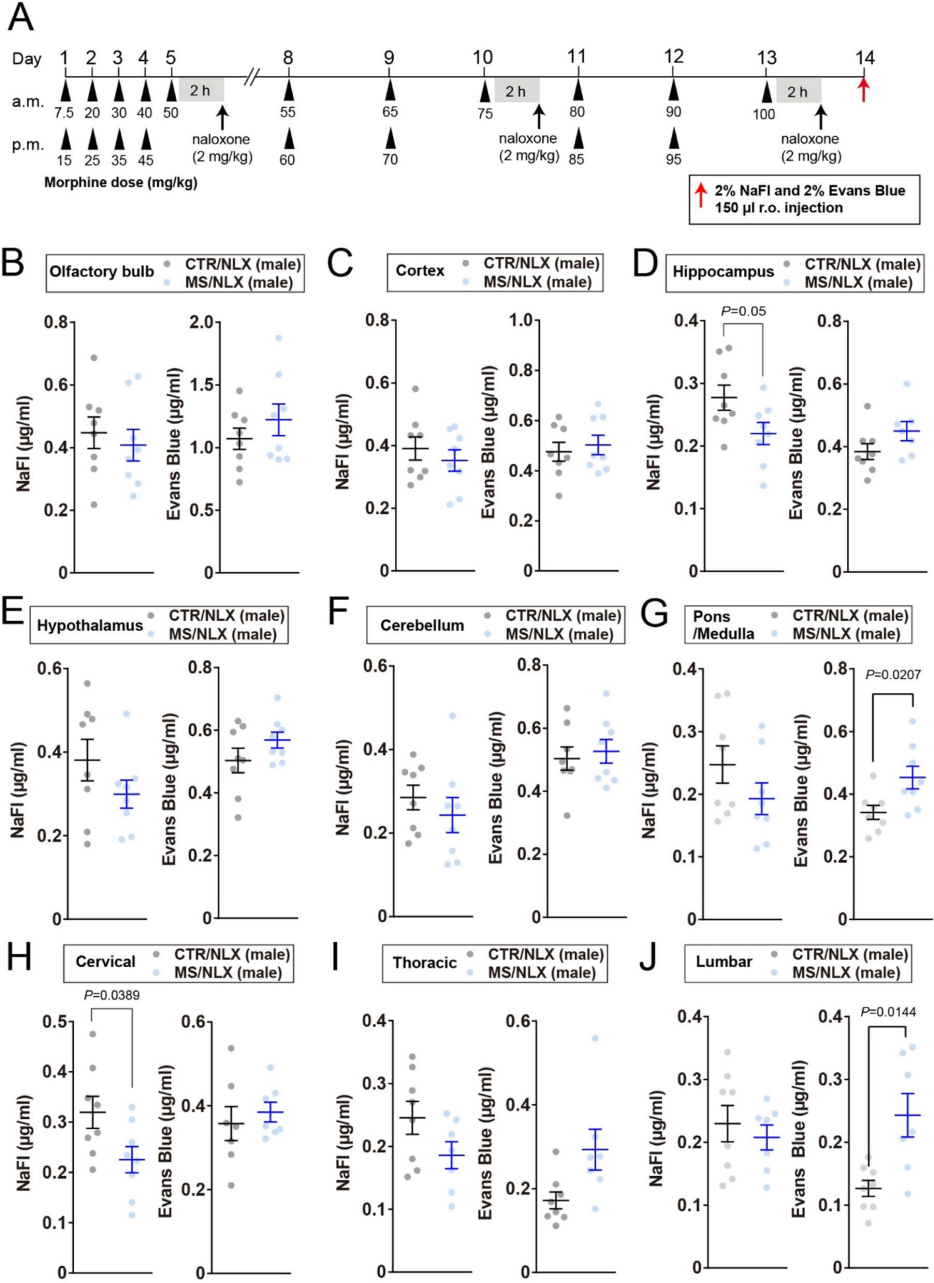
Naloxone challenge causes divergent NaFl and Evans Blue extravasation in pons/medulla and spinal cord of males. **A**, Schematic depicting morphine dosing, naloxone precipitated morphine withdrawal, and injection of dye tracers. Dye tracers are retro-orbitally injected 24 h after last morphine withdrawal. **B-J**, NaFl and Evans Blue extravasation into the olfactory bulb (**B**), cortex (**C**), hippocampus (**D**), hypothalamus (**E**), cerebellum (**F**), pons/medulla (**G**), cervical (**H**), thoracic (**I**) and lumbar (**J**) of male mice after third naloxone challenge (*n* = 7–8 mice). Data show the mean ± SEM

**Fig. 8:**
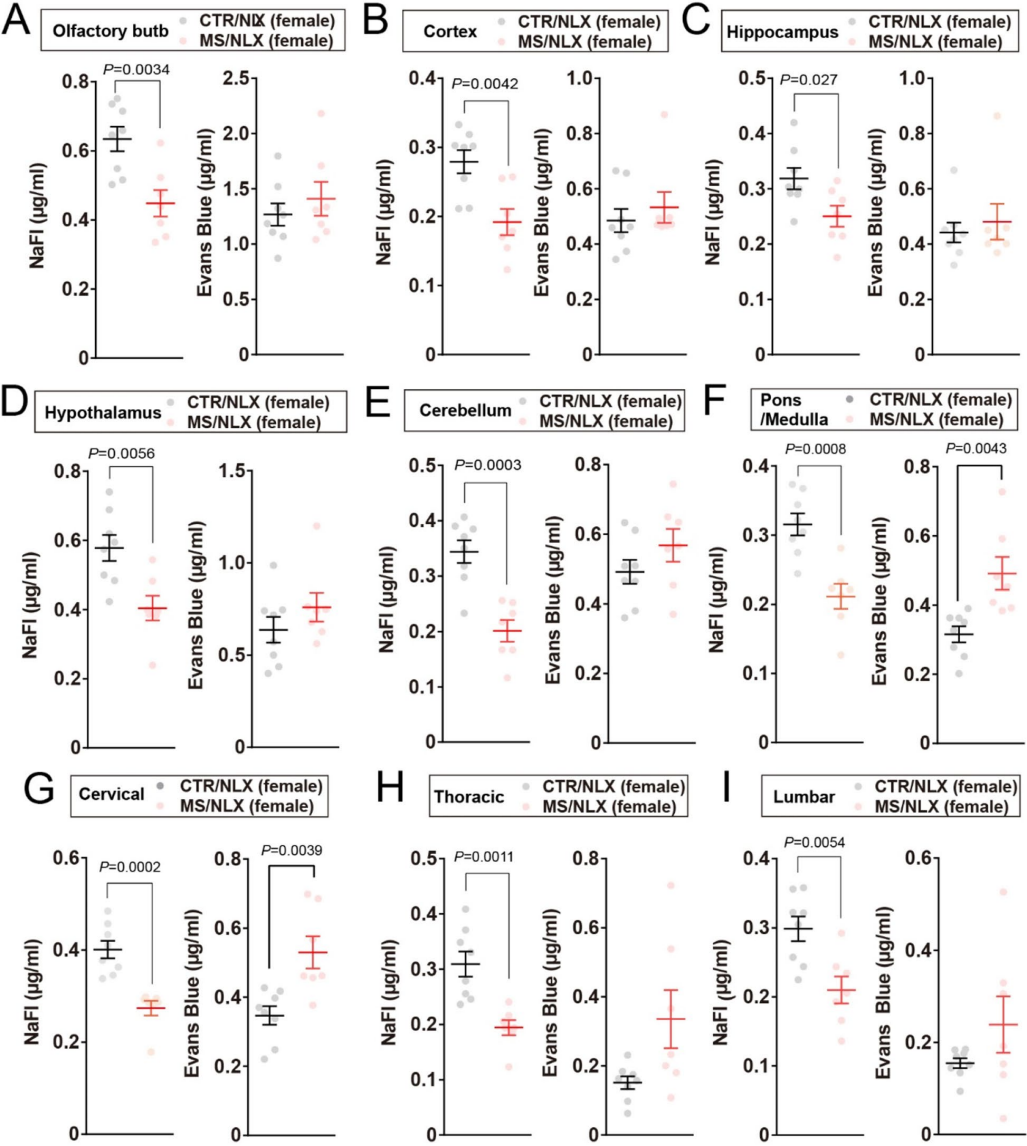
Naloxone challenge causes divergent NaFl and Evans Blue extravasation in pons/medulla and spinal cord of females. **A-J**, NaFl and Evans Blue extravasation into the olfactory bulb (**A**), cortex (**B**), hippocampus (**C**), hypothalamus (**D**), cerebellum (**E**), pons/medulla (**F**), cervical (**G**), thoracic (**H**) and lumbar (**I**) of female mice 24 h after third naloxone challenge (*n* = 7–8 mice). Data show the mean ± SEM

To assess the effects of repeated naloxone precipitated withdrawal following escalating doses of morphine on BBB and BSCB permeability, mice were peripherally injected with Evans Blue and NaFl 24 h after the final withdrawal on day13 (Fig. 7A). Repeated withdrawal significantly increased Evans blue dye extravasation in the pons/medulla region of the brainstem in both sexes (Figs. 7G and 8F), as well as in the lumbar spinal cord of male mice (Fig. 7J) and the cervical spinal cord of female mice (Fig. 8G). Similar trends were observed in other regions of the spinal cord in both sexes (Figs. 7H, I and 8H, I). With regard to other regions such as the olfactory bulb, cortex, hippocampus, hypothalamus, and cerebellum, Evans Blue levels did not differ between morphine withdrawn and control mice in both sexes (Figs. 7B-F and 8A-E). By contrast, NaFl extravasation displayed a sex-dependent pattern. In female mice, repeated withdrawal significantly reduced NaFl permeability across all examined brain and spinal cord regions (Fig. 8A-I). In male mice, NaFl levels were unchanged, with the exception of reduced extravasation in the hippocampus and cervical spinal cord (Fig. 7D and H), while other regions remained unaffected (Fig. 7B, C, E–G, I and J). Collectively, these findings demonstrate that repeated withdrawal from escalating morphine doses alters the BBB and BSCB permeability in a size-selective- and sex-dependent manner, with increased permeability to large molecules and restricted permeability to small molecules observed predominantly in female mice.

## Discussion

In this study, we examined the effects of repeated withdrawal from a fixed dose- or escalating doses of morphine on BBB and BSCB permeability in male and female mice. We observed that both paradigms of naloxone-precipitated morphine withdrawal resulted in higher withdrawal scores in morphine-treated compared to saline-treated control mice. However, these scores declined over subsequent withdrawal episodes. The reasons for this reduction are unclear, as some behaviors, such as tremors and wet-dog shakes, remained elevated compared to controls, confirming the persistence of withdrawal symptoms. One possibility is that repeated behavioral testing influences specific withdrawal behaviors, such as jumping and teeth chattering. Previous studies have shown that daily hot plate tests in rats can reduce latency over time [21], and consecutive testing on the elevated zero-maze in mice increases anxiety-like behaviors [22]. Additionally, intermittent opioid exposure and episodic withdrawal are known to promote psychomotor sensitization [23] and impair cognitive performance through increased apoptosis in the cortex and hippocampus [24]. These findings suggest that both repeated withdrawal and the testing paradigm may modulate the observed behavioral outcomes.

Our dye tracer experiments revealed that both repeated morphine withdrawal paradigms increased Evans Blue permeability in discrete regions of brain and spinal cord. Evans blue, which binds albumin, may reflect changes in albumin permeability mediated by endocytosis or transcytosis mechanisms [25, 26]. A recent study found that astrocyte-specific deletion of *Smo* (encoding Smoothened, a sonic hedgehog signaling transducer) transiently enhanced endocytosis and transcytosis without affecting paracellular boundaries [27]. We surmise that repeated morphine withdrawal could impact astrocyte-mediated BBB regulation, particularly in the medulla.

In contrast, NaFl extravasation was decreased in the all region of female mice and the cervical cord and hippocampus of male mice with repeated withdrawal from escalating doses of morphine. NaFl is commonly used tracer for assessing paracellular integrity, and their increased permeability is associated with disruptions in tight junction proteins or reduced pericyte coverage, as seen in neurodegenerative diseases [25, 26]. A previous report showed that loss of claudin-5, a critical tight junction protein, differentially affects permeability of small molecules (< 800 Da) like NaFl and larger molecules such as Evans Blue–albumin complexes [28]. Although in vitro studies suggest that morphine reduces claudin-5 expression [29], chronic morphine exposure did not alter claudin-5 levels in the hippocampus in vivo [30]. Whether repeated morphine withdrawal alters claudin-5 expression in other brain or spinal regions remains unknown and is an important direction for future research.

Sex differences were observed in the effects of repeated withdrawal from both paradigms of morphine treatments on BBB and BSCB permeability. There is evidence that male rodents may exhibit greater BBB vulnerability under conditions, such as traumatic brain injury, where reductions in occludin and zonula occludens-1 expression has been reported in males [31]. In contrast, sphingosine-1-phosphate receptor 2 has been identified as a sex- and strain-specific modulator of BBB permeability in female rodent models of experimental autoimmune encephalomyelitis [32]. Additionally, recent studies have demonstrated sex-specific alterations in BBB integrity following social stress in both mice and humans [33–36]. These findings suggest that sex may be a biological variable influencing the response of the BBB and BSCB, with effects potentially varying based the specific challenge or disease.

A key finding of this study is the identification of the medulla as a region particularly sensitive to BBB disruptions following repeated morphine withdrawal. This brainstem area includes the preBötzinger complex, which is critical for respiratory control [37], and the nucleus ambiguus, which regulates motor and autonomic functions [38]. These areas of the ventral medulla are enriched with mu opioid receptors, making them highly relevant targets of opioid action [39]. Despite their physiological significance, regional differences in BBB and BSCB function remain poorly characterized [40]. For example, a recent study using *pdfg-b*^ret/ret^ mice, which display pericyte loss, reported region-specific increases in Evans Blue permeability [41]. These differences are likely shaped by the molecular diversity of endothelial cells and their interactions with surrounding cell components. The median eminence, for instance, naturally lacks continuous endothelial tight junctions and exhibits a high BBB permeability to facilitate neuroendocrine signaling [42].

Collectively, our findings demonstrate that repeated morphine withdrawal alters the permeability of the BBB and BSCB in discrete regions of brain and spinal cords, highlighting regions specific vulnerabilities the central nervous system. Importantly, comparison of two morphine dosing paradigms suggests that repeated withdrawal, rather than dosing pattern alone, is a critical factor in barrier disruption. Future research will focus on how opioid exposure and repeated withdrawal affect key components of the neurovascular unit, including endothelial cells, pericytes, and astrocytes. It will also be important to investigate whether similar changes in barrier integrity occur with other opioids, such as fentanyl and heroin, to determine whether these effects are unique to morphine or generalizable across opioids. Identifying and targeting specific components of the neurovascular unit could offer promising strategies to mitigate BBB and BSCB dysfunction during repeated opioid withdrawal. Such approaches may help reduce the neurocognitive and systemic effects associated with repeated withdrawal and improve treatment outcomes for opioid use disorder.

## Methods

### Animals

Male and female C57BL/6 mice (aged 8 − 10 weeks, Charles River) were housed under a 12-h light/dark cycle with ad libitum access to food and water. Procedures were approved by the University of Calgary, in accordance with the Canadian Council on Animal Care guidelines.

### Morphine dosing paradigms

Morphine sulfate (PCCA Corp., London, ON, Canada) and naloxone hydrochloride dihydrate (Sigma, MO, USA) were dissolved in 0.9% sterile saline solution. To model acute and repeated opioid withdrawal, various morphine dosing and naloxone challenge paradigms were used in this study.

#### Acute naloxone-induced morphine withdrawal

Mice received intraperitoneal (i.p.) morphine twice daily (AM and PM, 8-h apart). Morphine was administered at escalating doses: day 1, 7.5 and 15 mg/kg; day 2, 20 and 25 mg/kg; day3, 30 and 35 mg/kg; and day 4, 40 and 45 mg/kg. On the morning of day 5, mice received an i.p. injection of 50 mg/kg morphine. Control mice received an equal i.p. volume of 0.9% sterile saline at all time points. Two hours after the last morphine or saline injection, naloxone (2 mg/kg; i.p.) was injected to rapidly induce morphine withdrawal [43, 44].

#### Repeated morphine withdrawal (a fixed dose of morphine)

Two days after a single morphine withdrawal, mice received a fix dose of morphine (50 mg/kg) on every morning from day 8 to day 17. Naloxone challenges (2 mg/kg; i.p.) were conducted everyday 2 h after morphine treatment on days 8–17.

#### Repeated morphine withdrawal (escalating doses of morphine)

Two days after a single morphine withdrawal, mice received ascending doses of morphine twice daily (day 8, 55 and 60 mg/kg; day 9, 65 and 70 mg/kg; day11, 80 and 85 mg/kg; day12, 90 and 95 mg/kg). On the morning of day 10 and day 13, mice received a morphine injection of 75 mg/kg (day 10) or 100 mg/kg (day 13) and 2 h later naloxone.

### Behavioral assessment of naloxone precipitated withdrawal

Signs of morphine withdrawal were recorded with some modification [43]. Jumping, headshakes, wet-dog shakes, licking, teeth-chattering, and tremors were recorded at 5-min intervals for a total test period of 30 min and a standardized score of 0 − 3 was given for each 5 min interval. In addition, the presence/absence of piloerection and tremors was scored every 5 min and given one point. All signs were added to yield a cumulative withdrawal score.

### Analysis of blood brain barrier and blood spinal cord barrier permeability

Under 2% isoflurane anesthesia, 2% sodium fluorescein (NaFl, Sigma, MO, USA) and 2% Evans blue (Sigma, MO, USA) (total 150 µl) dissolved in saline were injected retro-orbitally (r.o.) in mice based on a previous report with some modification [45]. Forty-five minutes later, mice were overdosed with isoflurane and transcardially perfused with phosphate buffer saline. Brain (olfactory bulb, cortex, hippocampus, hypothalamus, and pons/medulla) and spinal cord (cervical, thoracic, and lumbar cord) were snap-frozen on dry ice and stored at − 80ºC until use. Endogenous protein was extracted by trichloroacetic acid (TCA) according to a previous report [46]. Dye tracers were quantified in TCA-clarified lysate diluted 1:4 in 95% ethanol, with readings at 480 nm excitation/520 nm emission (NaFl) and 620 nm excitation/680 nm emission (Evans Blue) on a Gemini XPS or Spectra Max i3x (Molecular Device, San Jose, CA, USA) [45, 46].

### Statistical analysis

Statistical analysis was performed using Prizm 6 (GraphPad). Quantitative data were expressed as the means ± SEM. Data were analyzed by unpaired *t*-test (Figs. 3B-J, 4, 7B-J and 8), Mann–Whitney test (Fig. 3C, D (NaFl)), unpaired *t*-test with Welch’s correction (Figs. 7I, J and 8H and I (Evans Blue)), and repeated measures two-way ANOVA with the post hoc Bonferroni’s multiple comparisons test (Figs. 1B-I, 2, 5B-I and 6), as appropriate, after determining the normality (Shapiro–Wilk test) and variance (F-test) of the experimental data. Values were considered significantly different at *P* < 0.05.

## Data Availability

The dataset used and/or analyzed during the current study are available from the corresponding author on reasonable request.

## Abbreviations

BBB: Blood-brain barrier
BSCB: Blood-spinal cord barrier
CNS: Central nervous system
NaFl: Sodium fluorescein
